# Nano-Zinc Iron Oxide Elicits Oxidative Stress-Driven Midgut Damage and Developmental Suppression in *Bombyx mori*

**DOI:** 10.3390/biology15191731

**Published:** 2026-10-01

**Authors:** Zhen He, Jiaying Lin, Bing Chen, Liang Huang, Cui Yu, Hui Ai, Fan Wu

**Affiliations:** 1 Industrial Crops Institute, Hubei Academy of Agricultural Sciences, Wuhan 430064, China; zhenhe_nky@hbaas.com (Z.H.);; 2 College of Life Sciences, Central China Normal University, Wuhan 430079, China

**Keywords:** zinc iron oxide, *Bombyx mori*, midgut pathology, oxidative stress, metabolism pathway

## Abstract

The silkworm, *Bombyx mori*, a key economic insect, has been employed as a model organism to investigate the toxicological effects and underlying mechanisms of zinc iron oxide nanoparticle exposure on its growth and development, midgut tissue structure, and physiological functions. Exposure to zinc iron oxide inhibited larval growth and development, which was accompanied by pathological alterations in the midgut, including a reduction in columnar cells, vacuolation, chromatin condensation, microvilli shedding, and disrupted tight junctions, which directly impaired the intestinal barrier integrity and nutrient absorption. Furthermore, zinc iron oxide exposure resulted in elevated reactive oxygen species levels, decreased superoxide dismutase and glutathione contents, and increased catalase activity, indicating the activation of oxidative stress. This stress-induced mitochondrial response, characterized by the observation of autophagic vesicles, suggests the potential mitophagy of activation in action to zinc iron oxide exposure, thereby preserving cellular homeostasis. This was also associated with the downregulation of most genes involved in purine metabolism, the peroxisome, the biosynthesis of amino acids, the ribosome pathway, and carbon metabolism pathways, leading to reduced protein synthesis and energy supply, ultimately resulting in larval developmental retardation. This study elucidates the intestinal-targeted toxicity of zinc iron oxide nanoparticles in lepidopteran insects, providing direct in vivo experimental evidence for evaluating the ecological risk of zinc iron oxide in sericulture environments. Furthermore, it offers a novel research avenue for developing intestinal intervention strategies aimed at mitigating the toxic effects of nanomaterials and ensuring the stability of economically important traits in silkworms.

## Simple Summary

The silkworm, *Bombyx mori,* serves as a model organism to study the effects of zinc iron oxide nanoparticles on its growth, development, and midgut structure. Zinc iron oxide exposure hinders larval growth, causes midgut damage, and disrupts nutrient absorption. It elevates reactive oxygen species, reduces antioxidant levels, and triggers oxidative stress. These likely triggers stress-induced mitophagy to maintain cellular homeostasis. This study highlights the intestinal toxicity of zinc iron oxide nanoparticles in lepidopteran insects, offering in vivo evidence to assess zinc iron oxide’s ecological risk in sericulture. It also opens new research paths for intestinal interventions to reduce nanomaterial toxicity and maintain key traits in silkworms.

## 1. Introduction

The expanding utilization of zinc iron oxide (ZnFe_2_O_4_) within biomedical science, industrial manufacturing, environmental remediation, and other high-value sectors has significantly increased the probability of their introduction into natural ecosystems through atmospheric deposition, hydrological runoff, and solid waste leaching pathways [1,2,3,4]. Consequently, the biosafety and ecotoxicological characteristics of these engineered nanomaterials have become a critical yet underexplored priority in current environmental toxicology research. Unlike well-characterized single-metal oxide nanomaterials such as nano-ZnO and nano-Fe_3_O_4_, ZnFe_2_O_4_ are spinel-structured bimetallic nanocomposites whose biological effects cannot be directly inferred from the combined toxicity of their monometallic counterparts. Their distinctive crystalline lattice configuration, exceptionally high specific surface reactivity, and pH-dependent ion release kinetics collectively result in more complex in vivo metabolic pathways and modes of toxicological action compared to conventional single-phase nanomaterials.

Previous toxicological studies utilizing aquatic model organisms and mammalian cell lines have rigorously demonstrated that ZnFe_2_O_4_ nanoparticles, at elevated exposure concentrations, are capable of traversing biological membrane barriers [5,6,7,8]. These nanoparticles preferentially accumulate in the epidermal cells and systemic circulatory system, where they trigger an excessive intracellular production of reactive oxygen species (ROS) [5]. This overproduction disrupts the homeostatic balance of cellular antioxidant defense systems, subsequently inducing a cascade of adverse effects, including oxidative stress, cytotoxicity and apoptosis [5,6]. Nevertheless, the majority of current toxicological data is predominantly concentrated on aquatic ecological compartments, such as the Mediterranean mussel and in vitro cellular assays [5,6], resulting in a significant knowledge gap regarding the systematic toxicity assessment of zinc iron oxide in terrestrial holometabolous insects. Insects, being the most species-rich taxon within terrestrial ecosystems, demonstrate significant vulnerability to exogenous nano-contaminants throughout their feeding, growth, and metamorphic processes. These toxic effects have the potential to propagate through trophic levels, ultimately disrupting material cycling and energy flow across the entire terrestrial food web.

The domestic silkworm, *Bombyx mori*, serves as a quintessential lepidopteran model organism that is distinguished by its well-annotated reference genome, standardized mass-rearing protocols, and quantifiable developmental endpoints [9,10,11,12]. These attributes render it an ideal in vivo model for the toxicological evaluation of exogenous nanomaterials in terrestrial insects. Numerous studies have previously demonstrated that dietary exposure to certain metal-based nanomaterials can lead to dose-dependent alterations in silkworm development [11,12,13,14,15]. However, systematic investigations into the toxicological effect of zinc iron oxide in silkworms remain unexplored.

Within this research framework, the current study employed a gradient dietary exposure approach to systematically investigate the toxicological effects and underlying mechanisms of zinc iron oxide on the growth and development of silkworms. This research not only address a significant gap in the toxicological profiling of spinel-structured bimetallic nanomaterials in terrestrial insects but also provides essential invertebrate toxicity data to inform the tiered ecological risk assessment of zinc iron oxide. Furthermore, it offers empirical theoretical guidance for mitigating the potential hazards posed by emerging nano-contaminants in sericultural ecosystems, thereby contributing to the long-term sustainability of the global silk industry.

## 2. Materials and Methods

### 2.1. Silkworm Strain and Rearing

Newly ecdysed silkworm larvae of the Chufeng × Hanyun strain (Wuhan, China) were obtained from the Industrial Crops Institute at the Hubei Academy of Agricultural Sciences in Wuhan, P.R. China. The fifth-instar larvae, selected for their uniform body size, were maintained under controlled conditions with a 12 h light–dark cycle at a constant temperature of 25 ± 1 °C and a relative humidity of 70–75% in accordance with established protocols [10]. Each larva at the fifth-instar stage was individually housed in a separate Petri dish, and all dishes were collectively placed in a constant-temperature incubator. The silkworm experiences four molting cycles, during which it sheds its exoskeleton and undergoes progressive growth. The interval between each molting cycle is termed an “instar”. Therefore, a silkworm that has completed four molts is classified as being in its fifth instar.

### 2.2. Zinc Iron Oxide Nanoparticles Treatment

Commercial zinc iron oxide (ZnFe_2_O_4_, CAS No.12063-19-3) nanoparticles were procured from Macklin Biochemical Co., Ltd. (Shanghai, China), which were characterized by a primary particle size of 50 nm and a purity level ≥ 99.5%. An independent characterization of particle morphology, size distribution, hydrodynamic diameter, zeta potential, crystallinity and elemental composition under the present exposure conditions was not performed in this study. Prior to the preparation of the exposure system, the material was pre-dispersed in ultrapure water and homogenized using an ultrasonic processor. Ultrasonication was conducted utilizing a 200 W probe sonicator under ice-bath conditions with the amplitude maintained at 30% for a continuous duration of 15 min. Throughout the procedure, the temperature of the dispersion was meticulously regulated to remain below 25 °C to prevent any potential alterations in the surface properties of the nanoparticles due to elevated temperatures. Healthy mulberry leaves, sourced from the garden, were submerged in zinc iron oxide suspensions at concentrations of 0.4, 0.8,1.6, 3.2, and 6.4 μg/μL for a duration of 10 min to ensure a uniform coating of the leaf surface with suspension to ensure that the adhesion conditions of nanoparticles on the surface of each mulberry leaf are completely consistent, which is consistent with previous study [11]. Fresh nanoparticle dispersions were prepared daily immediately prior to use. Prior to immersing the mulberry leaves, the dispersion was thoroughly homogenized through gentle agitation, ensuring that no visible agglomeration or sedimentation was evident before the exposure procedure. Subsequently, the leaves were allowed to air-dry at room temperature for 1 h to eliminate residual surface water. In the control group, mulberry leaves were subject to the same immersion and air-drying procedures using ultrapure water without the addition nanoparticles. We implemented an isolated single-individual rearing protocol, wherein each silkworm of the same instar was provided with equal and adequate quantities of treated mulberry leaves thrice daily at 08:00, 14:00, and 20:00. This approach was designed to ensure consistent exposure while minimizing leaf spoilage and nanoparticle sedimentation. Prior to each subsequent feeding, any remaining mulberry leaves and deceased larvae were promptly removed with the residual leaves being weighed and documented. Additionally, the number and body weight of deceased larvae were recorded concurrently before the first feeding of each day.

### 2.3. Determination of Antioxidant Indicators

Healthy fifth-instar silkworm larvae on day 5 were selected for use as experimental subjects. Each group consisted of three biological replicates with each replicate containing four larvae. The larvae were thoroughly ground into a powder using a pre-cooled mortar with liquid nitrogen. Subsequently, 500 μL of pre-cooled 1× phosphate-buffered saline (PBS) was added to the samples from both groups. The resulting mixture was vortexed thoroughly to produce tissue homogenates. These homogenates were then centrifuged at 8000g and 4 °C for 10 min, after which the upper clear supernatant was carefully collected for the subsequent analysis of antioxidant-related indicators. All procedures were conducted on ice to prevent the inactivation of enzyme activity. 

To determine ROS level, the oxidation-sensitive probe, 2, 7-dichlorofluorescein diacetate (DCFH-DA, Applygen, Beijing, China), was employed for detection purposes. A volume of 30 µL of the pretreated supernatant was diluted to 100 µL using PBS, followed by the addition of 100 µL of DCFH-DA working solution, achieving a final concentration of 30 µmol/L. The mixture was thoroughly homogenized and subsequently transferred to an opaque 96-well fluorescent microplate. Incubation was conducted at 37 °C in the absence of light for a duration of 30 min. Fluorescence intensity was measured using a FLx 800 fluorescent microplate reader (Bio-Tek, Winooski, VT, USA) with the excitation wavelength set at 485 nm and the emission wavelength at 520 nm. The resultant ROS level was quantified and expressed as reactive oxygen species units per milligram of protein (U/mgprot).

For the determination of SOD activity, the nitroblue tetrazolium (NBT) colorimetric assay was employed for detection purposes. A sequential addition of 18 µL of the test supernatant, 48 µL of PBS, 12 µL of xanthine oxidase, 36 µL of 1.25mmol/L NBT working solution, 6 µL of xanthine, and 68 µL of double-distilled water as performed into a 96-well microplate. Following thorough mixing, the mixture was incubated at 37 °C for 30 min. Absorbance readings at a wavelength of 560 nm were obtained using a microplate reader, and SOD activity was determined based on the inhibition rate with results expressed in U/mgprot. In the context of CAT assessment, the ammonium molybdate termination colorimetric method was employed for detection. A volume of 20 µL of the test supernatant was introduced into 100 µL of a hydrogen peroxide (H_2_O_2_) substrate solution at a concentration of 20 µmol/mL, and the mixture was incubated precisely at room temperature for 10 min. Subsequently, 180 µL of 50 mmol/L ammonium molybdate tetrahydrate was added to promptly terminate the reaction. The absorbance was measured using a microplate reader set to a detection wavelength of 405 nm. CAT activity was calculated based on the reaction rate of CAT decomposing hydrogen peroxide with a result expressed in U/mgprot. For the GSH content, the 5,’5-dithio-bis (2-nitrobenzoic acid) (DTNB) colorimetric method was employed. A volume of 0.5 mL of the test supernatant was combined with 2 mL of the precipitant provided with the kit. Following thorough mixing, the mixture was subjected to centrifugation at 3500 g for 10 min. Subsequently, 1 mL of the resulting supernatant was used for the colorimetric reaction. Reagent buffer, DTNB chromogenic solution, and substrate working solution were added sequentially. After allowing the mixture to stand at room temperature for 5 min, absorbance was measured at a wavelength of 412 nm using a microplate reader. The GSH concentration in the sample was calculated using a standard curve, and the results were expressed in U/mgprot. 

The activities of SOD, CAT, and GSH were evaluated utilizing detection kits procured from the Nanjing Jiancheng Bioengineering Institute (Nanjing, China), adhering strictly to the manufacturer’s protocol. During the assessment of all indicators, blank control wells and standard calibration wells were established. The intra-assay coefficient of variation was maintained below 2% to ensure the repeatability and reliability of the detection outcomes.

### 2.4. Histopathological Examination

The fifth-instar larvae were anesthetized utilizing an ice-cooling method, after which the intact midgut tissues were meticulously dissected and isolated. These tissues were subsequently rinsed with pre-cooled 0.01M phosphate-buffered saline (pH 7.4) to remove any residual intestinal contents, which was followed by fixation in 10% neutral-buffered formalin at 4 °C for a period of 12 to 24 h. The tissues underwent a series of processing steps, including dehydration through a graded ethanol series, clearing with xylene, infiltration with paraffin, and embedding. They were then sectioned into slices of 4–6 μm thickness using a microtome and baked at 60 °C for 2 h to ensure firm adhesion to poly-L-lysine-coated glass slides. The sections were deparaffinized in xylene and rehydrated through a graded ethanol series. Staining was performed with hematoxylin for 5–8 min, which was followed by rapid differentiation in 1% hydrochloric acid alcohol for 2–3 s. The sections were then blued in a weakly alkaline solution, rinsed with running water, and counterstained with eosin for 1–2 min. After re-dehydration, and xylene clearing, the sections were mounted with neutral balsam. Finally, the tissue sections were examined and imaged using a Nikon DS-U3 imaging system (Nikon, Tokyo, Japan).

### 2.5. Transmission Electron Microscopy (TEM)

On the fifth day, four fifth instar from both the ZIO-treated (administrated at a concentration of 6.4 µg/µL) and the control group were anesthetized using CO_2_. Subsequently, their midgut tissues were swiftly isolated and immediately fixed in 2.5% glutaraldehyde at 4 °C overnight. The pre-fixed tissues underwent thorough rinsing with 0.2 mol/L phosphate buffer (pH 7.4) and were then post-fixed in 1% osmium tetroxide (OsO4) for 1 h. Following this dual fixation process, the samples were dehydrated through a graded ethanol series and embedded in Araldite (EMbed 812, Beijing, China). Ultrathin sections, with a thickness of 60–70 nm, underwent double staining with 2% uranyl acetate and lead citrate for 10 min. Subsequently, these sections were examined and imaged using a JSM-2100 transmission electron microscope (JEOL, Tokyo, Japan). The microscope was operated at an accelerating voltage of 200kV, utilizing a Schottky thermal field emission electron gun, and employed in parallel bright-field imaging mode. The magnification range was adjusted from ×2000 to ×200,000 to facilitate the high-resolution visualization of subcellular ultrastructural pathological alterations in the silkworm midgut, such as chromatin condensation, mitochondrial cristae damage, and endoplasmic reticulum dilation.

### 2.6. RNA-seq and Bioinformatics Analysis

On the fifth day, four fifth instar from the group treated with a concentration of 6.4 µg/µL zinc iron oxide nanoparticles, along with those from the control group, were anesthetized using CO_2_. Total RNA was extracted employing RNAiso Plus (Takara, Dalian, China) in accordance with the manufacturer’s instructions. The concentration of the extracted total RNA was measured using a Nanodrop ND-2000 (Thermo Fisher Scientific, Waltham, MA, USA), and RNA integrity was evaluated using an Agilent Bioanalyzer 4150 system (Applied Biosystem, Foster City, CA, USA). Paired-end libraries were meticulously constructed following the ABclonal RNA-seq Lib Prep Kit (ABclonal, Wuhan, China). This process involved the enrichment and isolation of mRNA using oligo-dT magnetic beads, which was followed by the fragmentation of mRNA and its reverse transcription into cDNA using SuperScript II reverse transcriptase (Transgen, Beijing, China). Indexed Illumina adapters were then ligated to the cDNA fragments, and limited-cycle PCR was performed. The PCR products were purified with the AMPure XP system (Beckman Coulter, CA, USA), and the quality of the libraries was re-assessed with the Agilent Bioanalyzer 4150 (Agilent Technologies, CA, USA). Finally, 150 base paired-end reads were generated using the DNBSEQ^TM^ platform (BGI Genomics, Shenzhen, China). 

HISAT2 (version 2.2.1) was employed to align the sequencing reads to the *Bombyx mori* reference genome (BmDZ.v2.6.gff3). HTSeq was utilized to count raw read counts per gene, representing the unnormalized gene-level expression values. These raw read counts were used as the input matrix for DESeq2 differential-expression analysis. Gene-expression values for descriptive visualization were quantified using Reads Per Kilobase of transcript per Million mapped reads (RPKM). Differentially expressed genes (DEGs) were identified based on a log2(fold change) threshold of ≥1 and a *q*-value of <0.05 across three biological replicates by Differential Expression analysis for Sequence data 2 (DESeq2, https://bioconductor.org/packages/release/bioc/html/DESeq2.html, accessed on 28 April 2026) (Package version, 1.52.0). DESeq2 performs internal size-factor normalization for statistical testing, and RPKM values were not used for DEG statistical inference. A False Discovery Rate (FDR) < 0.05 was employed to regulate the false positive rate. Following this, Kyoto Encyclopedia of Genes and Genomes (KEGG) pathway analysis was conducted using clusterProfiler R to evaluate the statistical enrichment of DEGs within the relevant biological pathways. Benjamini–Hochberg correction was applied for multiple testing, and pathways with adjusted *p*-values (FDR) < 0.05 were defined as significantly enriched.

### 2.7. Reverse Transcription Quantitative Transcriptase-Polymerase Chain Reaction (RT-qPCR)

On the fifth day, four fifth instar from the group treated with zinc iron oxide at a concentration 6.4 μg/μL, along with those from the control group, were anesthetized using CO_2_. Total RNA was extracted utilizing the Trizol reagent (Invitrogen, Carlsbad, CA, USA) in accordance with the manufacturer’s instructions. All procedures were conducted on a pre-cooled, RNase-free workbench to prevent RNA degradation. The concentration and purity of extracted RNA were assessed using a Nanodrop 2000 spectrophotometer, and samples exhibiting an A260/A280 ratio between 1.9 and 2.1 were selected for further analysis. Subsequently, 1 μg of the qualified total RNA served as the template for reverse transcription, which was performed using the reverse transcriptase (EasyScript, TransGen, Beijing, China) and oligo dT18 primer under the conditions of 37 °C for 30 min followed by 85 °C for 5 min to stop the reaction. The synthesized cDNA was stored at -20 °C for future use. Specific primers were designed based on sequences obtained from sequencing results (Table S1). An RT-qPCR assay was conducted using a CFX connect^TM^ real-Time System (Bio-Rad, Hercules, CA, USA) in conjunction with the SYBR Green qPCR Mix (TransGen). The reaction mixture, with a total volume of 20 μL, compromised 10 μL 2 × SYBR Premix, 0.5 μL of 10 μM forward primer, 0.5 μL of 10 μM reverse primer, 2 μL of diluted cDNA template and 7.2 μL of RNase-free water. The amplification protocol was initiated with a denaturation step at 95 °C for 3 min, which was followed by 40 cycles of amplification at 95 °C for 5 s, 60 °C for 30 s, and 72 °C for 20 s. Subsequently, a melting curve analysis was conducted over a temperature range of 55 °C to 98 °C. The silkworm housekeeping gene *Actin 3* was employed as the internal reference for normalization purposes, and the relative gene expression levels were determined using the 2^−ΔΔCT^ method. Each sample was analyzed in triplicate for technical replicates, and three independent biological replicates were performed to ensure the robustness and reliability of the experimental outcomes [16,17]. The primers used in this experiment are shown in Table S2.

### 2.8. Protein–Protein Interaction

The PPI analysis was performed via the STRING database (version 12.0) with a minimum interaction-confidence combined score of 0.7 (high confidence). The network was imported into Cytoscape (version 3.10.4) for topological visualization and module analysis. Degree centrality was calculated using the CytoHubba plugin (version 0.1), and nodes were ranked and arranged according to their degree values. The highly connected nodes were then grouped into functional modules based on their annotation. Two principal hub modules were identified: a purine/one-carbon-folate metabolism module comprising SHMT, GART, GLDP, and ADSL, which aligns with the purine metabolic alterations, and a ribosomal protein module, which was further extracted and visualized as a separate subnetwork. 

### 2.9. Statistical Analysis

Data were expressed as mean ± SEM. A two-way ANOVA was conducted using Graphpad Prism (version 11.0) to compare the control and experiment group for the larval growth experiment. A two-tailed Student’s *t*-test was performed using GraphPad Prism (version 11.0) for all experiments except the larval growth experiment. Survival differences among the treatment groups were statistically evaluated using the log-rank test. Statistical significance was defined as a *p*-value of less than 0.05.

## 3. Results

### 3.1. Effects of Zinc Iron Oxide on Silkworm on Larval Development

**Figure 1:**
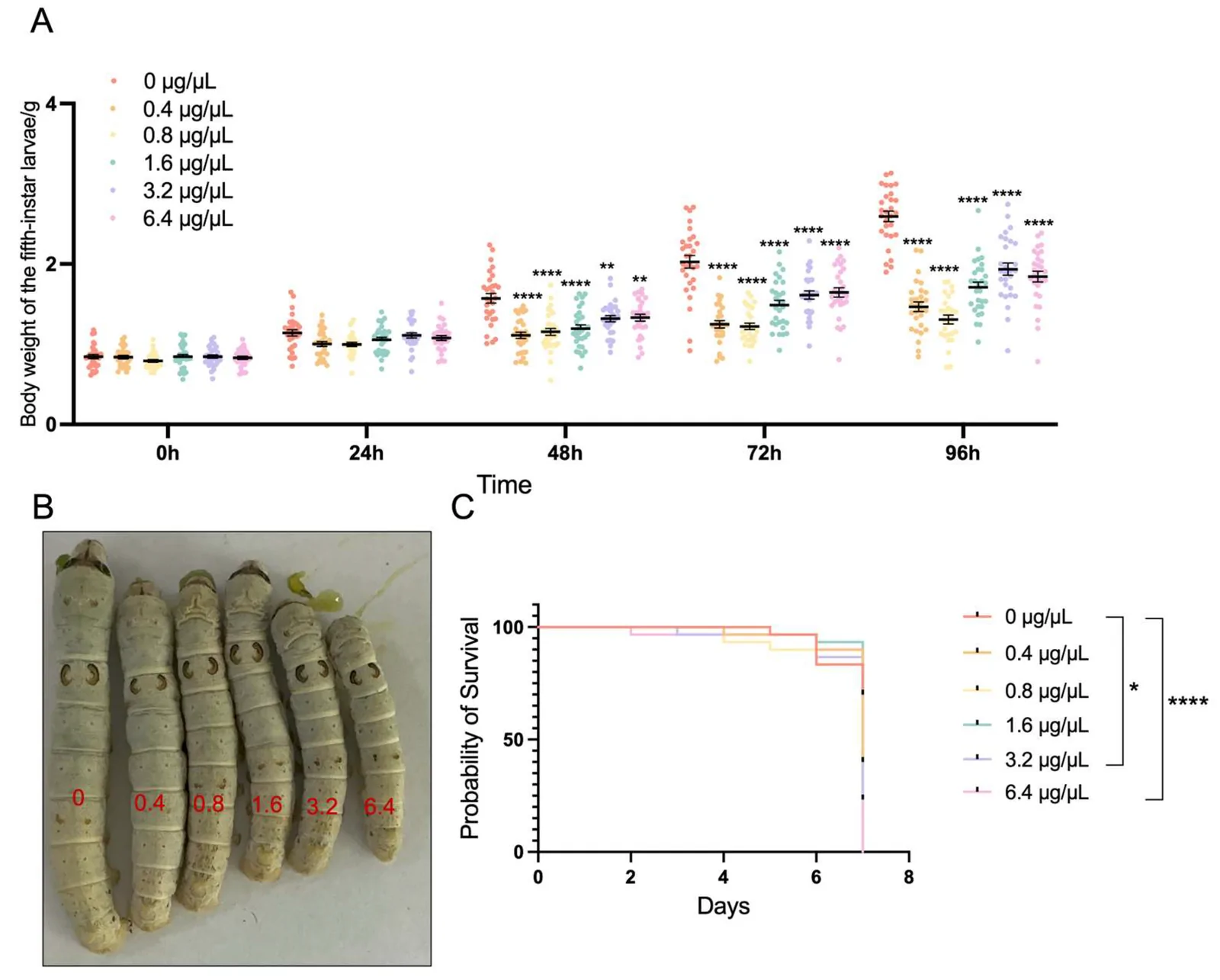
Developmental progression of silkworm larvae subjected to different concentrations of zinc iron oxide. (**A**) Daily weight measurement of individual fifth-instar larvae (*n* = 30). (**B**) Morphological observed on the fourth day of the fifth-instar stage. (**C**) Survival rates of fifth-instar larvae exposed to varying concentrations of zinc iron oxide. Data were presented as mean ± SEM. Statistical significance is indicated by “*”: *p* < 0.05, “**”: *p* < 0.01, and “****”: *p* < 0.0001.

The study investigated the effects of zinc iron oxide nanoparticles exposure on the growth of silkworm larvae with particular emphasis on changes in larval body weight as a primary indicator of developmental progress. Fifth-instar larvae were provided with mulberry leaves treated with various concentrations of zinc iron oxide, and the weight of each larva was recorded at intervals of 0, 24, 48, 72, and 96 h (*n* = 30). Daily weight measurements of the fifth-instar larvae commenced on the first day of this development stage following supplementation with different concentrations of zinc iron oxide nanoparticles (Figure 1A). Initially, no significant differences in weights were observed between the experimental and control groups (Figure 1A). From the second day of the fifth instar onwards, the body weight of larvae in all treatment groups exposed to ZIO at varying concentrations was significantly reduced compared to the control group during the same period (Figure 1A). Additionally, it was noted that the larval size on the fourth day of the fifth instar decreased with increasing concentrations of zinc iron oxide (Figure 1B). Additionally, the larval mortality was also assessed over a duration of 1 to 7 days (Figure 1C). The survival rate of the control group was 76.67%. In contrast, the survival rates for larvae exposed to concentrations of 0.4 μg/μL, 0.8 μg/μL, 1.6 μg/μL, 3.2 μg/μL, and 6.4 μg/μL were 40%, 40%, 43.33%, 26.67%, and 0%, respectively, by day 7, when the larvae were preparing to spin cocoons (Figure 1C). The observed survival rate of 76.67% in the control group is noteworthy, as it falls well within the anticipated range of variation for individually reared fifth-instar silkworm larvae during the autumn season. This survival rate does not undermine the validity of the comparative analysis of toxicological effects between the experimental and control groups.

### 3.2. Zinc Iron Oxide-Induced Oxidative Stress in Silkworm Larvae

**Figure 2:**
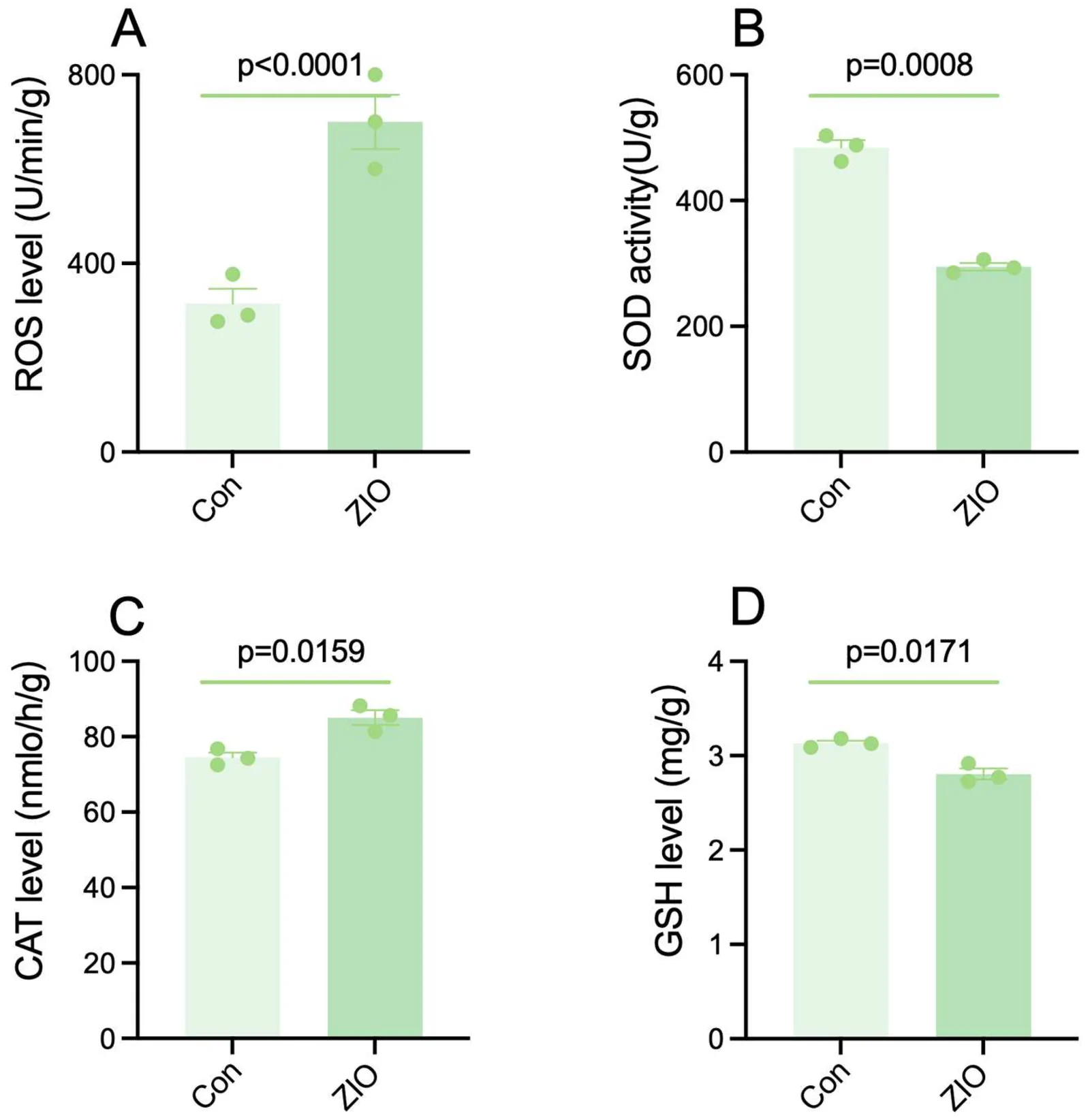
Analysis of oxidative stress in fifth-instar silkworm larvae subjected to zinc iron oxide exposure. (**A**) ROS levels were significantly elevated following exposure to ZIO. (**B**) SOD activity was markedly reduced in ZIO-treated silkworm larvae. (**C**) CAT showed a significant increase upon ZIO exposure. (**D**) GSH levels were significantly decreased in silkworm larvae treated with ZIO. *n* = 3, and data were presented as mean ± SEM.

To systematically evaluate whether exposure to zinc iron oxide induces oxidative stress in the silkworm, a comprehensive analysis of canonical oxidative stress-related biomarkers was conducted in the fifth-instar larvae. Given that the lifespan results indicated an extremely significant difference in mortality rates between the highest concentration and the control group, we chose the highest concentration for subsequent experiments. In fifth-instar larvae exposed to 6.4 μg/μL of zinc iron oxide (ZIO), there was a statistically significant increase in intracellular ROS levels compared to the untreated group, clearly indicating the onset of severe oxidative stress in vivo (Figure 2A). Simultaneously, the total activity of SOD, a crucial first-line antioxidant enzyme responsible for ROS scavenging, was significantly reduced under the same treatment conditions (Figure 2B). Although exposure to high concentrations of zinc iron oxide stimulated a compensatory upregulation of CAT activity as an adaptive antioxidant response (Figure 2C), this partial compensatory mechanism was insufficient to mitigate oxidative damage, as demonstrated by the significant depletion of reduced GSH levels (Figure 2D). These coordinated alterations of multiple redox biomarkers collectively provide compelling evidence that exposure to zinc iron oxide significantly activates the oxidative stress in silkworm larvae, thereby disrupting the intrinsic redox homeostasis between pro-oxidant generations and endogenous antioxidant defense mechanisms.

### 3.3. Zinc Iron Oxide-Induced Multidimensional Pathological Lesions in Silkworm Larval Midgut

**Figure 3:**
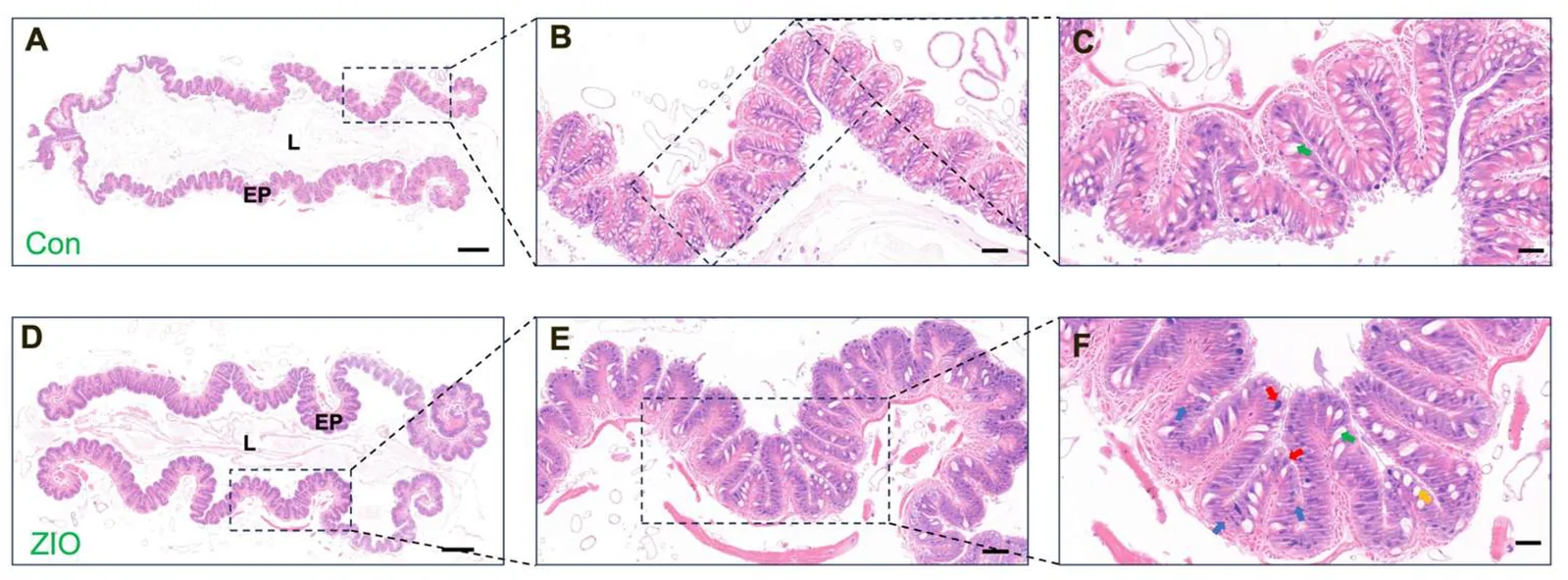
Histomorphological alterations in the midgut of silkworm larvae following exposure to zinc iron oxide. (**A**–**C**) The histomorphology of midgut in the control group. (**D**–**F**) The histomorphology of midgut in the ZIO-treated group. Scale bar as follows: 500 µm (**A**,**D**), 100 µm (**B**,**E**), 50 µm (**C**,**F**). Epithelium: EP, Lumen: L. The green arrow indicates columnar cells, the golden arrow signifies midgut cytoplasm vacuolization, the red arrow denotes chromatin condensation, and the blue arrow highlights distributed cytoplasmic eosinophilic granules.

To elucidate the histomorphological and structural variations in the silkworm midgut following exposure to 6.4 μg/μL of ZIO, hematoxylin–eosin (HE) histopathological staining was conducted on midgut tissues obtained from both the treatment group and the untreated control group, thereby enabling systematic observation and analysis. In contrast to the control group, which was not exposed to ZIO, the ZIO-treated group exhibited significant multidimensional pathological alterations in the midgut epithelial structure (Figure 3). Specifically, there was a marked reduction in the number of widely distributed columnar cells (indicated by green arrows), and epithelial cell vacuolation was observed (indicated by golden arrows) within the midgut epithelium compared to the control group (Figure 3C,F). Additionally, a significant increase in nuclear density was observed within the epithelial layer, characterized by the majority of nuclei exhibiting chromatin condensation, which manifested as an enlarged and plump morphology (Figure 3F, red arrow). Furthermore, abundant eosinophilic granules were distributed in the cytoplasm of the ZIO-treated silkworm larvae midgut (Figure 3F, blue arrow). These histopathological changes substantiate that exposure to ZIO at this concentration induces discernible damage to the normal homeostatic structure of the silkworm midgut epithelium, thereby compromising its structure integrity. 

### 3.4. Zinc Iron Oxide-Induced Ultrastructural Alterations in Silkworm Midgut Epithelium

Building on the previously discussed histopathological findings that ZIO exposure disrupts the homeostatic architecture of the silkworm midgut epithelium at the tissue level, we conducted an in-depth investigation into the subcellular pathological changes to acquire more precise and detailed evidence of the epithelial damage phenotype. To substantiate the multi-scale pathological impact of the ZIO on the silkworm midgut epithelium observed at the histological level, we employed transmission electron microscopy (TEM) to systematically characterize the subcellular ultrastructural alterations in the midgut epithelial cells of silkworm larvae.

**Figure 4:**
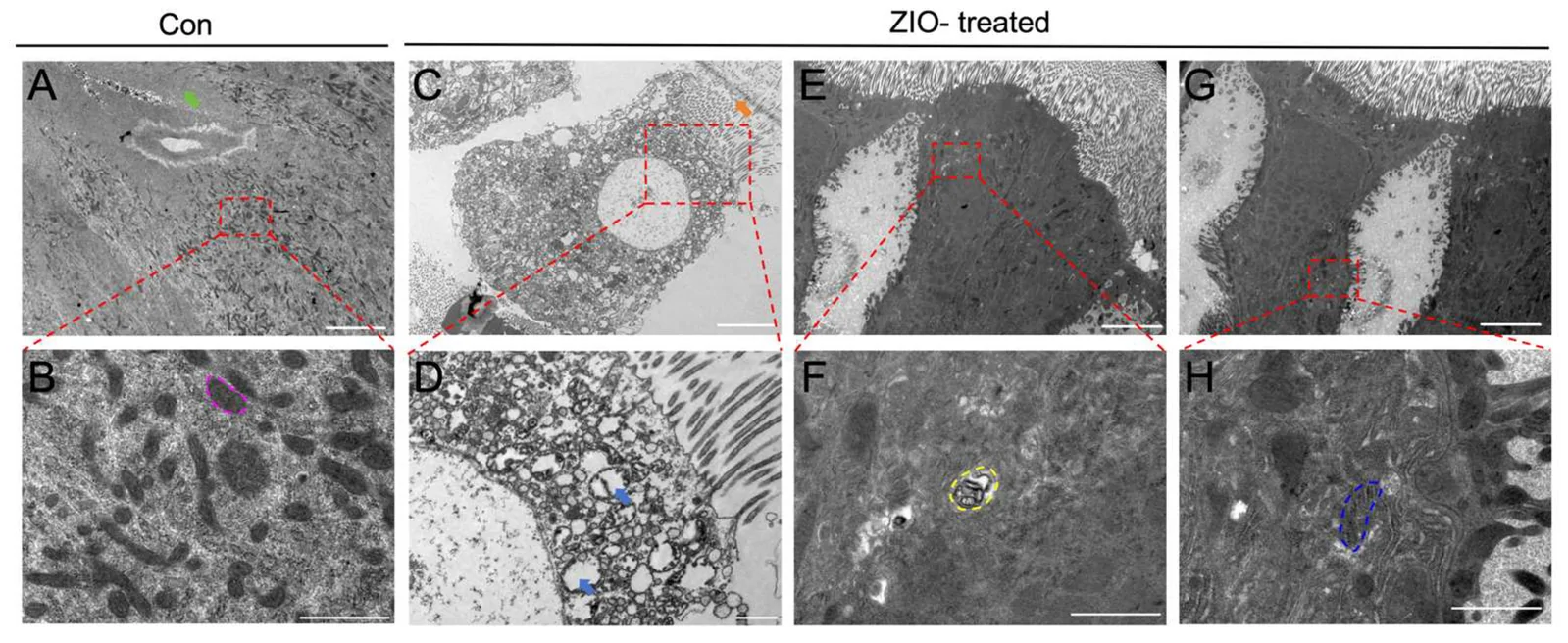
Ultrastructural analysis in silkworm midgut epithelial cells. (**A**,**B**) The ultrastructural observation in control group. (**C**–**H**) The ultrastructural observation in ZIO-treated group. Green arrow indicates normal microvilli. Blue arrows indicate cytoplasmic vacuoles, and orange arrows indicate microvilli lodging. Purple dashed lines mark intact mitochondria, yellow dashed lines mark autophagosomes eclosing damaged mitochondria, and blue dashed lines signify damaged mitochondria. Scale bar: 5 µm (**A**,**C**,**E**,**G**), 1 µm (**B**,**D**,**F**,**H**).

In the control group, the midgut epithelial cells exhibited optimal structural homeostasis. The mitochondria retained their intact double-membrane architecture with tightly and orderly arranged cristae, while the apical membrane of the epithelial cells was covered with a continuous, dense layer of microvilli that were evenly distributed and free from morphological defects (Figure 4A,B). However, following exposure to 6.4 μg/μL ZIO, the midgut epithelial cells displayed significant ultrastructural disruptions. Numerous cytoplasmic vacuoles of varying sizes were diffusely distributed within the cytoplasmic compartment, severely disrupting the normal spatial arrangement of functional organelles. The apical microvilli exhibited typical pathological phenotypes, including lodging, disarrangement, and partial exfoliation, which directly impaired the absorptive capacity and physical defense performance of the midgut. Furthermore, the mitochondrial structure underwent dramatic pathological remodeling, which was characterized by widespread mitochondrial swelling, blurred cristae boundaries, and extensive cristae rupture.

Significantly, a substantial number of autophagosome structures were observed in the ZIO-treated group, indicating the potential activation of mitophagy. This is evidenced by the autophagosomes encapsulating damaged mitochondria observed within the field of study. These ultrastructural findings at the subcellular level align closely with previously documented histopathological observations. This evidence confirms that ZIO exposure disrupts the normal homeostatic structure of the silkworm midgut epithelium and compromises its structural integrity.

### 3.5. RNA-seq Analysis of the Effect of Zinc Iron Oxide Exposure on Silkworm Gene Expression Profiles

To systematically analyze the transcriptomic regulatory mechanisms underlying the growth and the developmental changes in *B. mori* following exposure to zinc iron oxide, we conducted mRNA sequencing on samples from both the ZIO-treated group (ZIO) and the untreated control group (Con). As presented in Table 1, the ZIO-treated group produced between 36.85 and 55.43 million clean reads across all biological replicates, whereas the control group generated between 43.23 and 52.58 million clean reads. Notably, over 91.67% of the clean reads in all samples achieved Phred-like quality scores at the Q30 level, indicating a base-calling error probability as low as 0.001. The GC content of the sequencing libraries ranged from 44.49% to 44.93% for the ZIO group and from 44.68% to 46.76% for the control group (Table 1). Upon aligning clean reads to the published *B. mori* reference genome, the unique mapping rate for each individual sample ranged from 83.93% to 90.13% with the proportion of clean reads exhibiting multiple mapping locations on the reference genome being less than 9.73% across all samples (Table 1). Collectively, these stringent quality metrics confirm the high reliability and adequacy of the sequencing data.

**Figure 5:**
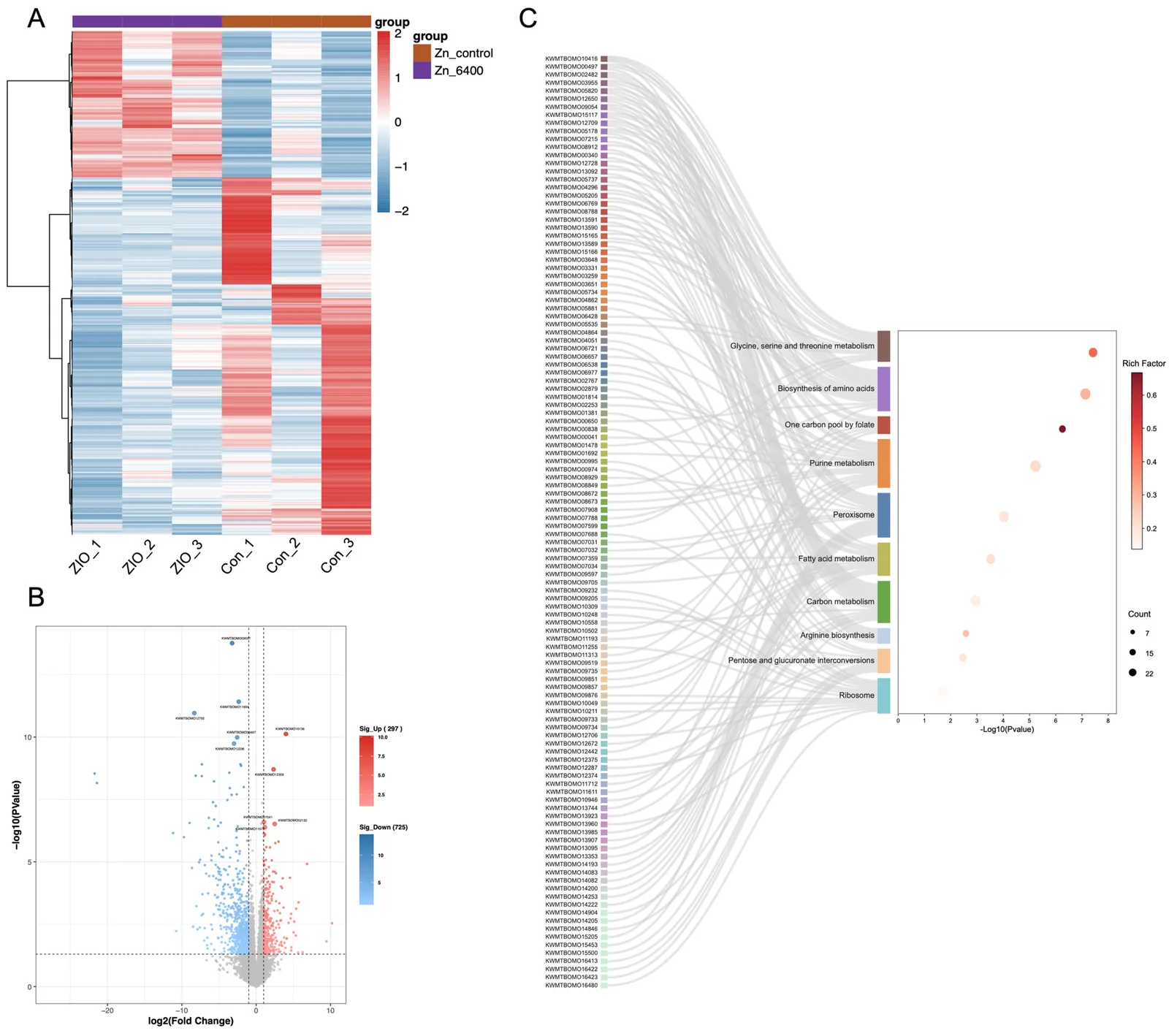
Alterations in mRNA expression determined by RNA-seq in silkworm larvae subjected to zinc iron oxide exposure. (**A**) A heatmap displayed the DEGs between the control and the ZIO-treated group. (**B**) A volcano plot visualizes the DEGs between the control and ZIO-treated group with red dots representing upregulated DEGs, blue dots indicating downregulated DEGs, and gray dots denoting unchanged DEGs. (**C**) The KEGG pathway analysis of the DEGs is conducted to identify and present the particularly enriched pathways. All pathways satisfied the threshold of adjusted *p*-value (FDR) < 0.05.

Following exposure to ZIO in the fifth-instar larvae, a total of 1,022 DEGs were identified using stringent screening criteria—specifically, an adjusted *q*-value < 0.05 and log_2_ (Fold Change) ≥ 1 (Figure 5A and Table S2), Among these DEGs, 297 genes were significantly upregulated, whereas 725 genes were significantly downregulated (Figure 5A and Table S2). This expression profile suggests that ZIO exposure primarily resulted in transcriptional repression rather that extensive transcriptional activation in silkworm larvae. 

To systematically analyze the potential biological functions and disrupted signaling pathways of these ZIO-responsive DEGs, we conducted KEGG pathway enrichment analysis. The analysis revealed that the purine metabolism pathway contained the highest number of mapped DEGs with 22 DEGs annotated to this pathway out of 233 pathway members. The peroxisome and biosynthesis of amino acids pathways were identified as the second most enriched pathways (Figure 5B, Table S3). Notably, significant enrichment was also observed in the ribosome pathway and carbon metabolism pathway (Figure 5C and Table S3), suggesting that ZIO exposure has a direct disruptive effect on the intracellular protein synthesis machinery and energy homeostasis in silkworm larvae. Overall, the pronounced enrichment of metabolism-related pathways in this analysis indicates that ZIO exposure primarily alters core metabolic regulatory networks in silkworm larvae. This finding is consistent with the oxidative stress and developmental retardation phenotype observed in previous experiments. 

According to the results of the KEGG pathway enrichment analysis (Table S3), three pivotal pathways were identified for further detailed investigation: purine metabolism, peroxisome, and ribosome pathways. These pathways demonstrated statistically significant enrichment, as indicated by low *p*-values, and exhibited relatively high rich factor values. The selection of these pathways for subsequent mechanistic exploration was further supported by the alignment between their functional annotations and the phenotypic changes observed in the preceding experiments.

### 3.6. Silkworm Purine Pathway Undergoes Reprogramming upon Zinc Iron Oxide Exposure

**Figure 6:**
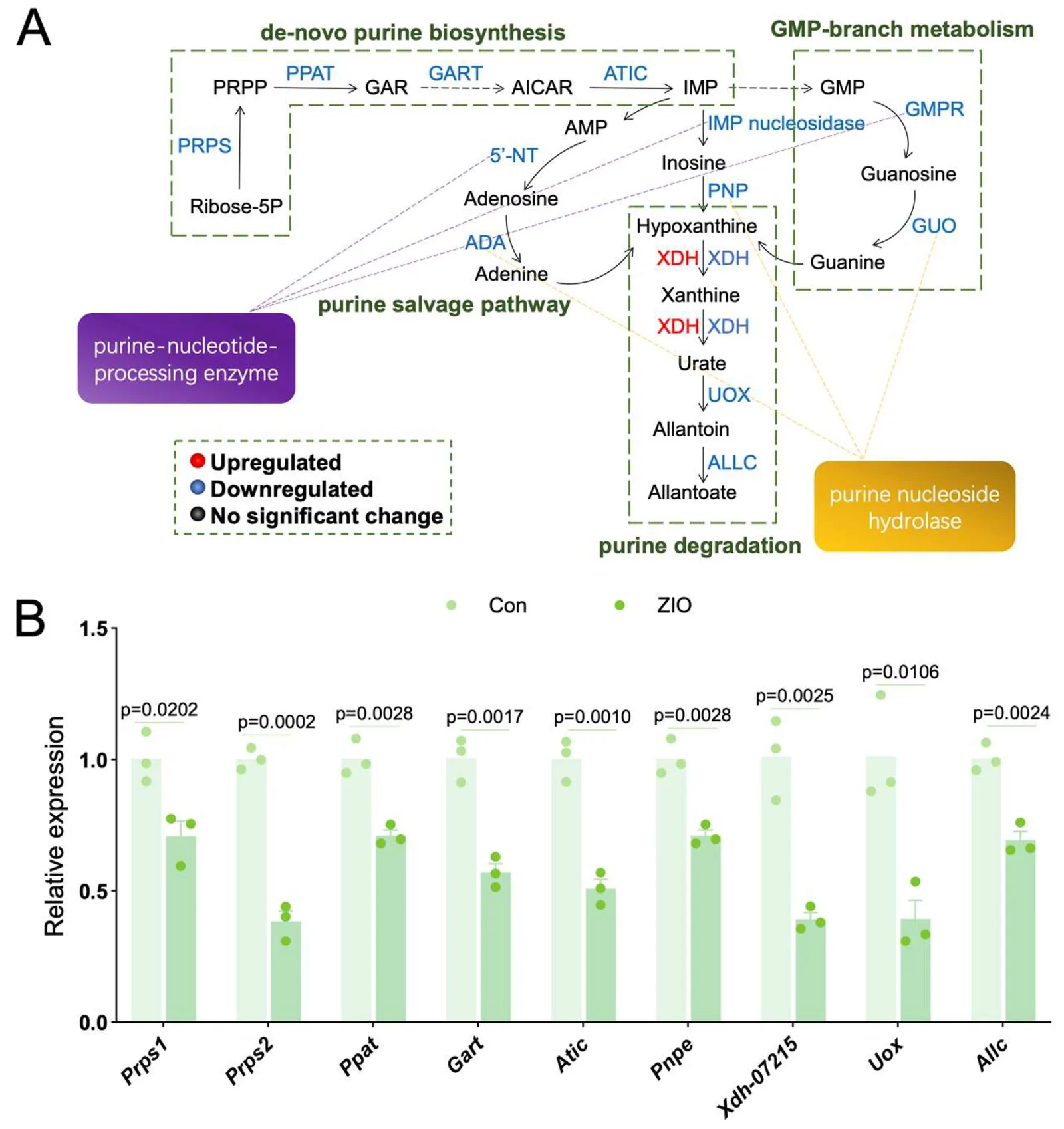
Diagram of the purine metabolism pathway in silkworm larvae under ZIO exposure and corresponding qRT-PCR validation. (**A**) Schematic representation of the perturbed purine metabolism pathway, encompassing de novo purine biosynthesis, the purine salvage pathway, GMP-branch metabolism, and purine degradation. Gene expression changes are indicated by font color: red denotes significantly upregulated genes, blue denotes significantly downregulated genes. 5′-NT, IMP nucleosidase and GMPR, connected by dashed lines, represent substrate-/reaction-dependent annotations of a single gene encoded purine-nucleotide-processing enzyme as per KEGG. Similarly, ADA, PNP and GUO, also connected by dashed lines, are substrate-specific annotations of another single-gene-encoded purine nucleoside hydrolase. (**B**) qRT-PCR validation of selected DEGs involved in the purine metabolism pathway is presented. The relative gene expression levels are compared between control (Con) and ZIO-treated silkworm groups with each dot representing an individual biological replicate. *n* = 3, and data were presented as mean ± SEM.

Purine metabolism serves as a pivotal hub in sustaining cellular energy homeostasis, nucleic acid metabolism, nitrogen metabolism, and redox balance. Its metabolic reprogramming may modulate insect growth, development, and metamorphosis [18,19]. To elucidate the molecular mechanisms underlying ZIO-induced developmental retardation in silkworms, we performed a KEGG pathway enrichment analysis based on transcriptomic data with a particular emphasis on global alterations in the purine metabolism pathway (Figure 6A). Pathway mapping revealed significant changes in the expression of numerous key genes involved in de novo purine synthesis, salvage pathways, GMP-branch metabolism, and purine degradation. Notably, the purine metabolism pathway was largely suppressed with the majority of DEGs being downregulated; only *Xdh-10699* exhibited an increase in expression (Figure 6A, Table S2). These findings suggest that ZIO exposure may induce a comprehensive remodeling of the purine metabolic network.

To rigorously validate the transcriptomic alterations, representative genes involved in purine metabolism were selected for qRT-PCR verification (Figure 6B). The qRT-PCR results exhibited a high degree of concordance with the transcriptomic data. Specifically, the genes *Prps1*, *Prps2*, *Ppat*, *Gart*, *Atic*, *Pnpe*, *Xdh-07215*, *Uox*, and *Allc* were significantly downregulated. Collectively, transcriptomic and qRT PCR data confirm the robust transcriptional suppression of purine metabolism-related genes upon ZIO exposure. Nevertheless, changes in transcript abundance cannot directly reflect in vivo purine turnover flux or actual metabolite levels. The widespread downregulation of key purine metabolic genes raises the hypothesis of transcriptional suppression of purine metabolism, which could potentially affect nucleotide pool homeostasis, compromise cellular energy conversion efficiency, and consequently disturb systemic energy balance. 

### 3.7. Zinc Iron Oxide Exposure Leads to Peroxisome Pathway Gene Alterations

Peroxisomes are highly integrated, and multifunctional metabolic organelles within eukaryotic cells. They function as a central platform for various catabolic pathways, including fatty acid β-oxidation, ether phospholipid synthesis, and amino acid catabolism. Additionally, they act as a crucial regulatory hub that balances ROS generation and scavenging, thereby playing an essential role in insect growth and development [20,21].

**Figure 7:**
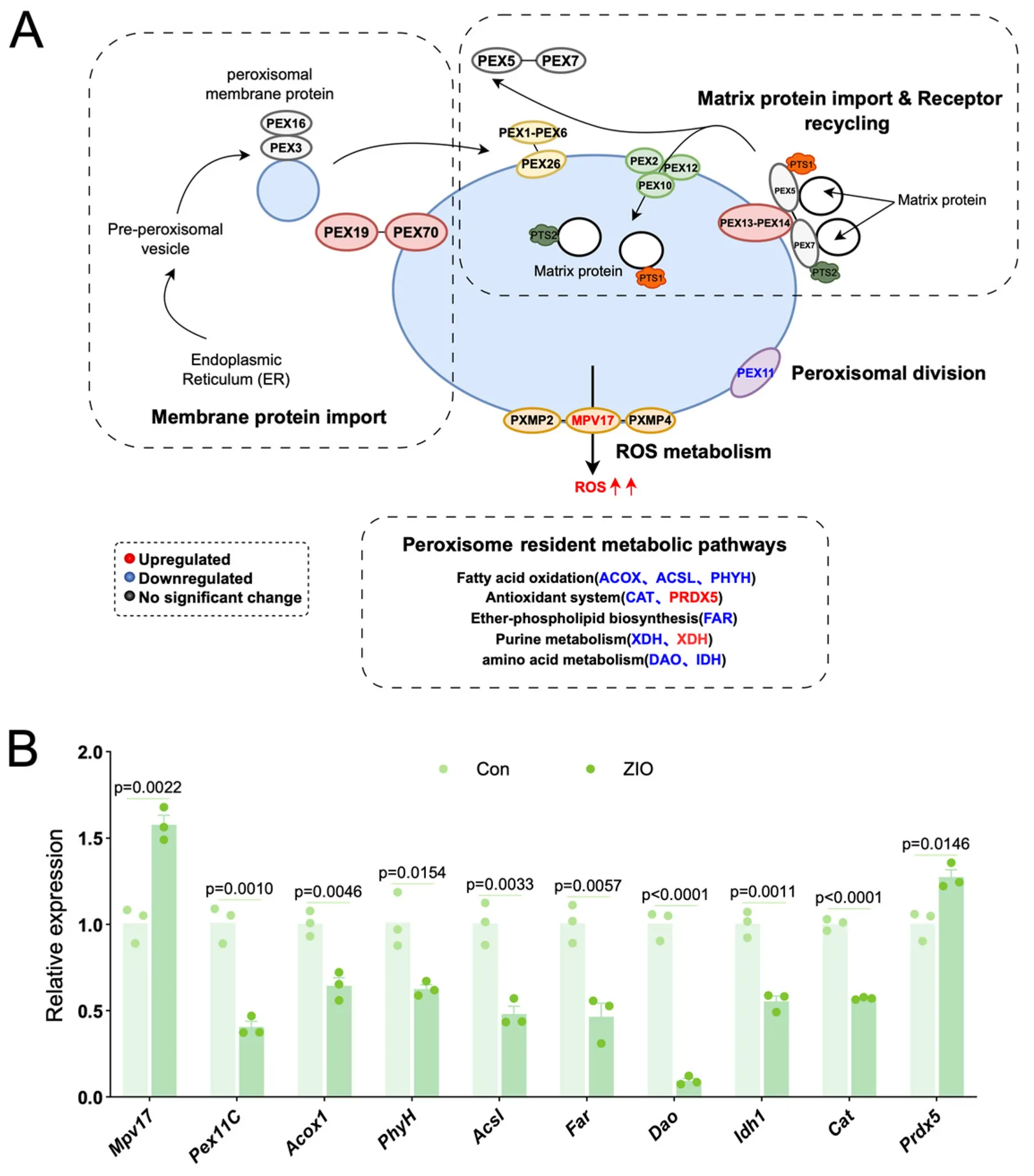
A schematic overview of molecular perturbations associated with peroxisomes induced by ZIO exposure in silkworm larvae is presented. (**A**) The diagram illustrates the disruptions caused by ZIO in peroxisome biogenesis, matrix protein import, receptor recycling, peroxisomal division, ROS metabolism, and resident metabolic pathways. Gene expression changes are indicated by font color: red denotes for significantly upregulated genes, blue indicates significantly downregulated genes, and black represents genes with no significant expression alteration. An increase in the ROS abundance is noted. The dashed box encapsulates the principal metabolic pathways within peroxisomes, including fatty acid oxidation, the antioxidant system, ether-phospholipid biosynthesis, purine metabolism, and amino acid metabolism. (**B**) Validation of representative peroxisome-related DEGs via qRT-PCR. Relative gene expression levels are compared between control (Con) and ZIO-treated silkworm groups with each dot representing an individual biological replicate. *n* = 3, and data were presented as mean ± SEM.

To elucidate the molecular mechanisms by which ZIO exposure disrupts energy homeostasis in silkworms from the perspective of metabolic–oxidative coupling, we constructed a schematic diagram of peroxisome-associated response pathways. This was based on KEGG enrichment analysis using transcriptomic data, systematically illustrating global transcriptional alterations in peroxisome biogenesis, protein import, organelle fission, ROS metabolism, and resident metabolic networks (Figure 7A). Under ZIO stress treatment, there were no significant changes in the transcription levels of genes related to peroxisome biogenesis and matrix protein import; the transcription levels of *Pex11c*, which regulates the homeostasis of organelle division, and *Mpv17*, which encodes the membrane permeability channel, were significantly altered; the genes encoding key enzymes in peroxisome function-related pathways, including fatty acid oxidation, ether phospholipid synthesis, amino acid metabolism, and the antioxidant defense system, exhibited extensive transcriptional downregulation characteristics. 

To validate these observations, we selected representative genes for qRT-PCR analysis (Figure 7B). The quantitative results were highly consistent with the transcriptomic trends: *Pex11c*, *Acox7*, *PhyH*, *Acsl*, *Far*, *Dao*, *Idh1*, *Cat*, and *Prdx6* were significantly downregulated, whereas *Mpv17* was significantly upregulated.

### 3.8. Silkworm Ribosome Pathway Exhibits Downregulation in Response to Zinc Iron Oxide Treatment

To elucidate the molecular basis underlying ZIO-triggered developmental delay in silkworms, a protein–protein interaction (PPI) network was constructed utilizing DEGs from significantly enriched KEGG pathways (*p* < 0.05). Topological analysis revealed two principal hub clusters. The first cluster, comprising SHMT, GART, GLDP and ADSL, predominantly involved purine and one-carbon-folate metabolism, aligning with the purine metabolic alterations discussed in Section 3.6 (Figure 6A). The second cluster primarily consists of ribosomal proteins, suggesting that the developmental abnormalities induced by ZIO stress in silkworms are closely linked to disruptions in protein synthesis (Figure 7A).

**Figure 8:**
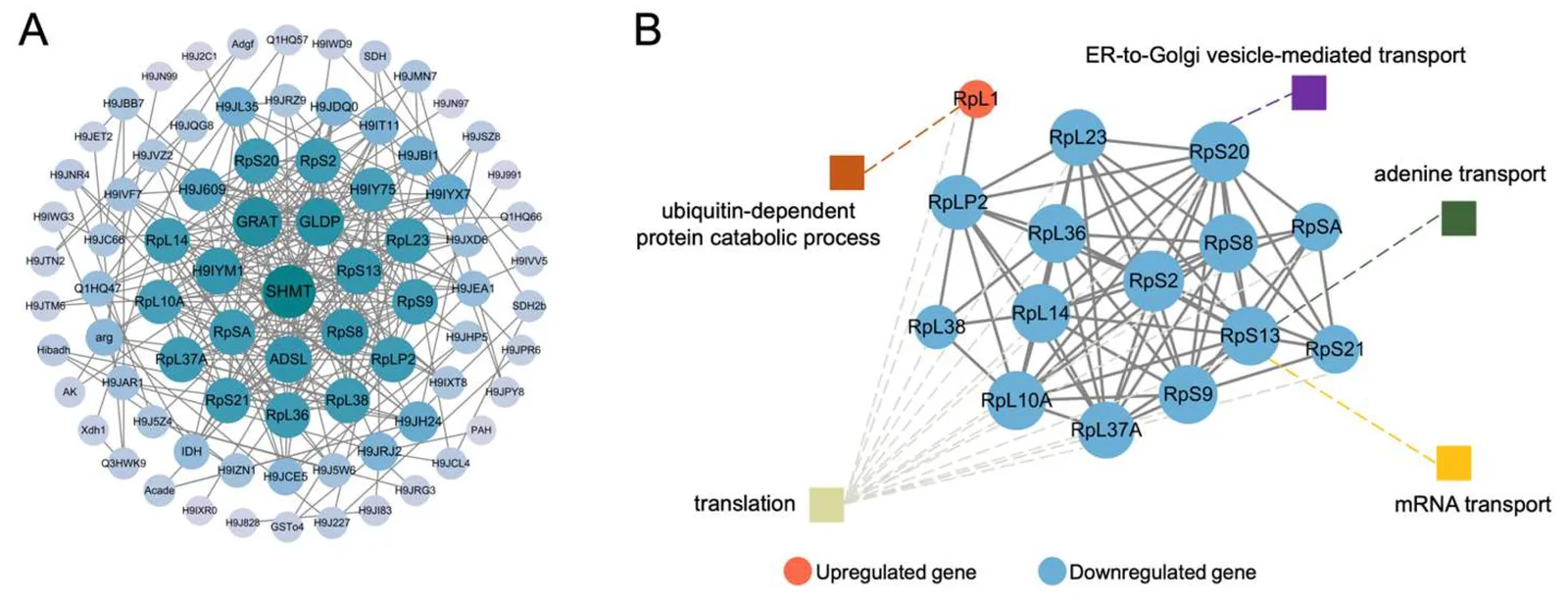
Protein–protein interaction (PPI) networks of differentially expressed genes following ZIO exposure in silkworm larvae. (**A**) PPI network constructed using DEGs annotated to KEGG pathways with enrichment *p* ≤ 0.05. The color and size of each node are scaled according to its degree value, while network edges indicate protein–protein associations. (**B**) PPI subnetwork of ribosome-associated DEGs. Red nodes represent significantly upregulated genes, and blue nodes represent significantly downregulated genes. Dashed lines connect genes to their corresponding enriched Gene Ontology terms, which include ubiquitin-dependent protein catabolic process, translation, ER-to-Golgi vesicle-mediated transport, adenine transport, and mRNA transport. Network edges signify protein–protein associations.

A ribosome-centered subnetwork was further visualized (Figure 8B). The colors of the nodes represent transcriptional upregulation or downregulation, while square nodes denote associated biological processes. Ribosomal proteins perform extensive pleiotropic functions, including roles in translation, mRNA transport, ER–Golgi vesicle trafficking, ubiquitin-dependent protein catabolism, and adenine transport. The majority of ribosomal proteins were downregulated, indicating a significant reduction in protein synthesis capacity. In contrast, RpL1 was uniquely upregulated. Beyond its structural role in ribosome assembly, RpL1 facilitates ubiquitin-dependent protein degradation, implying the simultaneous activation of protein catabolism under ZIO exposure. 

Through a comprehensive analysis of PPI networks, it is evident that exposure to ZIO triggers two primary physiological disruptions: a collapse in energy homeostasis derived from purine metabolism and an inhibition of ribosome-dependent protein synthesis. These disruptions are further compounded by hyperactivated protein degradation and an imbalance in peroxisomal redox states. Collectively, these physiological perturbations synergistically undermine normal cellular metabolism and developmental processes in the silkworm, ultimately manifesting as a developmental delay phenotype. 

## 4. Discussion

This study conducted a systematically investigation into the toxicological effects of zinc iron oxide nanoparticles on the growth and development of Bombyx mori, which is a well-established model insect in the Lepidoptera order. Most existing toxicological research predominantly employs aquatic model organism and mammalian in vitro cell lines, consistently demonstrating that zinc iron oxide, at elevated exposure levels, can traverse biological membrane barriers, induce excessive intracellular ROS production, and initiate oxidative damage [5,6]. However, terrestrial insects, which represent the most species-rich taxonomic group in terrestrial ecosystems, display significant species-specific differences in their response mechanisms to nanomaterials compared to aquatic organisms. Currently, a comprehensive toxicological assessment of zinc iron oxide in terrestrial holometabolous insects remains unexplored. Employing a gradient dietary exposure experimental design, this study investigated the toxicological effects of zinc iron oxide on silkworms by examining the underlying molecular mechanisms. This included an analysis of individual developmental phenotypes, the histopathological structures of the midgut, and the transcriptomic regulatory network. These findings offer critical in vivo experimental evidence from invertebrates for the tiered terrestrial ecological risk assessment. 

The results of our study demonstrate that the body weight of silkworm larvae in all treatment groups exposed to varying concentrations of ZIO nanoparticles was significantly reduced compared to the concurrent control group (Figure 1A). This finding provides direct evidence that ZIO nanoparticles exert a substantial inhibitory effect on the growth and development of the silkworms when administered through dietary exposure. Notably, larvae subjected to the highest concentration (6.4 μg/μL) of exposure failed to undergo metamorphosis and cocooning, resulting in a 100% cumulative mortality rate by the end of the fifth instar (Figure 1C). These observations collectively demonstrate that ZIO nanoparticles’ exposure induces a pronounced concentration-dependent lethal toxicity in silkworm larvae. Consistent with our findings, similar developmental retardation and reproductive suppression effects have been extensively documented in other species following exposure to different engineered nanomaterials [22,23,24,25]. For instance, in *Drosophila*, exposure to dietary silver nanoparticles has been demonstrated to markedly impair ovarian development in females and subsequently diminish reproductive fecundity [25]. These phenotype findings collectively provide a foundational basis for further investigation into the underlying mechanisms of intestinal toxicity and oxidative stress responses induced by ZIO nanoparticle exposure in silkworms.

At tissue levels, the midgut of silkworm, as the primary organ affected by the oral intake of exogenous pollutants, demonstrated a range of distinct pathological damage phenotypes when subjected to gradient dietary exposure to ZIO nanoparticles. These phenotypes include an extensive loss of columnar cells within the midgut epithelium, abnormal chromatin condensation within the nucleus, and a substantial accumulation of eosinophilic granules in the cytoplasm of epithelial cells (Figure 3). As the primary functional cell population in the silkworm midgut, which is integral to food digestion, decomposition, and trans-membrane nutrient transport, the substantial loss of midgut columnar cells suggests irreversible structural damage to the midgut’s core nutrient absorption function. Such histopathological alterations at the tissue level can be phenotypically correlated with the phenomena observed at the individual level: namely, the retarded growth and development of larvae and the significant reduction in body weight gain (Figure 1). Research indicates that the transmembrane dynamic of nanoparticles significantly influences their absorption within intestinal epithelial cells. The intracellular trafficking of nanoparticles, encompassing apical endocytosis, intracellular transport, and basolateral efflux, plays a crucial role in determining their bioavailability [26,27,28]. The disruption of this intricate intracellular process might lead to disturbances in intracellular substance transport pathways. In midgut epithelial cells, membrane-coated vacuolated structure are essential subcellular components that store digestive enzyme precursors during the larval stage of silkworms and facilitate intracellular vesicle trafficking and nutrient sorting [29]. Based on the transcriptomic and phenotypic observation results of this study, we speculate that exposure to high concentrations of ZIO may interfere with the substance transport pathways in midgut epithelial cells, thereby disrupting the original intracellular metabolic homeostasis. It should be noted that the histopathological evaluation in the present study was qualitative and based on a visual inspection of H&E-stained sections. Blinded semi-quantitative scoring and quantitative morphometric analysis were not performed. Future studies should incorporate blinded scoring and morphometric measurements to provide more objective evidence for epithelial injury, vacuolation, columnar-cell loss, and chromatin condensation.

Numerous prior studies have demonstrated that exposure to engineered nanoparticles can elicit substantial oxidative stress responses in living organism and human cells, as extensively documented nanoparticles such as ZnO, CuO, MgO, TiO_2_ and antimony tin oxide [11,13,14,30,31,32,33]. In alignment with these established findings, our research identified a similar oxidative stress phenotype in *Bombyx mori* following exposure to ZIO nanoparticles. Specifically, there was a significant increase in ROS levels in ZIO-treated larvae, a remarked decrease in SOD activity, a significant upregulation of CAT activity, and a significant reduction in GSH content (Figure 2). Transcriptomic sequencing analysis further revealed that the majority of DEGs in ZIO-exposed silkworms were downregulated with significantly enrichment in pathways related to purine metabolism, peroxisome, ribosome, and carbon metabolism. This transcriptional signature exhibits a distinct correlational relationship with the observed oxidative stress phenotype. Based on the above observations, we propose the following potential mechanistic hypothesis. The excessive production of ROS might disrupt the homeostasis of the antioxidant system, which may comprise SOD, CAT and GSH, thereby activating peroxisome-related stress pathways. Significant disturbances in the ribosome pathway might interfere with the synthesis of essential intracellular functional proteins, while inhibition of the carbon metabolism pathway disrupts cellular energy homeostasis. The synergistic effect of these disruptions might ultimately manifest as growth and developmental retardation in silkworms. Furthermore, the characteristic morphological features of nuclear pyknosis and abnormal chromatin aggregation observed in this study serve as histological markers indicating that midgut epithelial cells might enter apoptosis or necrosis (Figure 3F). These findings suggest that exposure to ZIO nanoparticles may initiate programmed cell death in midgut epithelial cells through upstream pathways such as oxidative stress. Subsequent molecular experiments will be required to verify our hypothesis.

In cellular systems, mitophagy plays a crucial role in maintaining mitochondrial quality control by removing dysfunctional mitochondria [34,35,36,37,38]. Subsequent ultrastructural analysis identified distinct pathological alterations, such as the dislodgement and shedding of midgut microvilli, mitochondrial swelling and rupture, and cytoplasmic vacuolization (Figure 4). Concurrently, the presence of autophagosome and mitophagy events suggests that the damaged mitochondrial have activated cellular quality control mechanisms. Prior research has established that mitophagy, within the context of biological effects induced by nanomaterials, is intricately linked to fluctuations in intracellular calcium levels, the excessive production of ROS, endoplasmic reticulum stress, and lysosome damage [39]. In alignment with these findings, our results have demonstrated a marked increase in ROS levels, suggesting that mitochondria likely initiate mitophagy in response to ZIO exposure to preserve cellular homeostasis. This observation aligns closely with previously documented intestinal targeted enrichment effects of nanomaterials in aquatic organisms [40,41,42,43,44] and elucidates the direct reason responsible for the disruption of midgut absorptive function and the physical defense barrier. 

As the application of ZIO continues to expand across various sectors, including biomedicine, industrial manufacturing, and environmental remediation, the risk of its environmental release into natural ecosystems is increasingly concerning. This release can occur through multiple pathways, such as atmospheric deposition and hydrological runoff [45,46,47]. The sericulture ecosystem represents a typical terrestrial agriculture scenario characterized by its sensitivity to the environment [10,11,12,13,48]; the ingestion of these particles by silkworms can lead to developmental retardation and cocooning failure [10,11]. This, in turn, results in a significant reduction in cocoon yield, thereby causing direct economic losses for farmers in sericulture production areas. This study elucidated the toxic effects of ZIO exposure on the growth and development of silkworms as well as its underlying mechanisms of action. The findings offer a fundamental theoretical basis for the formulation for targeted technical specifications for the prevention and control of nano-pollutants in major sericulture production regions. Additionally, this research provides essential toxicological data to support the long-term, sustainable development of the global silk industry.

## 5. Conclusions

**Figure 9:**
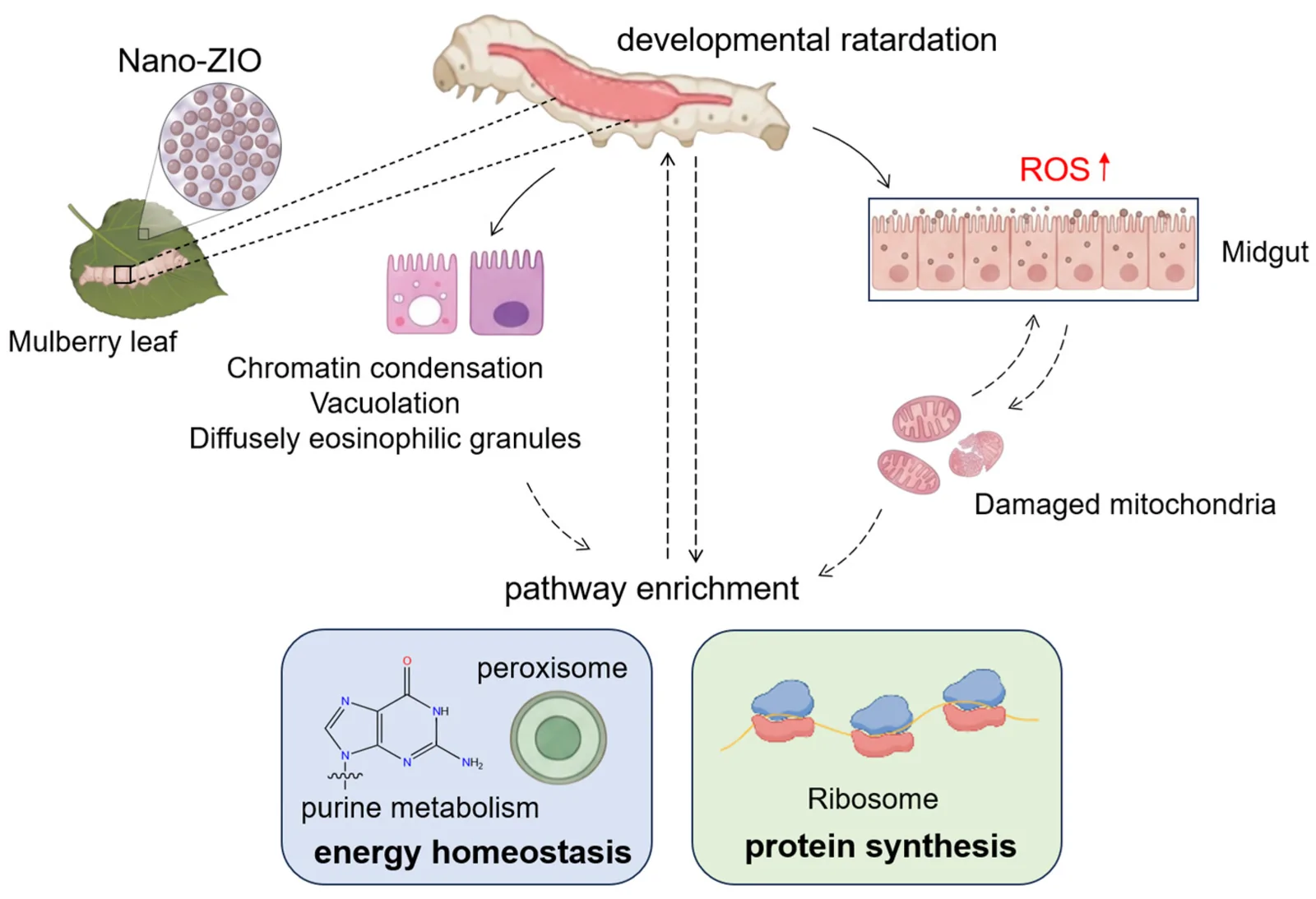
The schematic diagram in silkworm exposure to ZIO. ROS, Reactive Oxygen Species. Red arrow indicated overaccumulation of ROS.

In summary, our research demonstrated that exposure to ZIO adversely affects larval development by impairing growth and causing midgut damage. It led to an increase in ROS and a concomitant decrease in antioxidant levels, thereby initiating oxidative stress (Figure 9). Future research should focus on the targeted functional validation of the key pathways identified through the differential enrichment analysis of the purine metabolism, peroxisome, amino acid biosynthesis, ribosome, and carbon metabolism pathways conducted in this study. This will facilitate a more detailed examination of the direct interaction mechanisms between ZIO nanoparticles and core toxicity-responsive genes, thereby elucidating their specific molecular targets in lepidopteran insects, such as *B. mori.* Additionally, it is imperative to conduct a chronic toxicity assessment of the generational transmission in silkworms subjected to prolonged exposure to low concentrations of ZIO. This will enhance the comprehensive ecological risk assessment framework for iron-based nanomaterials within the sericulture ecosystem.

## Figures and Tables

**Table 1:** Summary of transcriptome sequencing data.

Samples	Con_1	Con_2	Con_3	ZIO_1	ZIO_2	ZIO_3
Clean reads	45,484,454	43,231,402	52,578,485	55,432,055	36,852,178	46,035,752
Q30 of clean reads	91.67%	92.57%	93.44%	92.45%	92.90%	93.57%
GC contents	46.76%	44.68%	45.36%	44.93%	44.49%	44.51%
Total mapped reads	42,310,528 (93.66%)	41,407,864 (96.49%)	50,569,436 (96.87%)	53,150,196 (96.53%)	34,988,704 (95.61%)	43,926,636 (96.16%)
Uniquely mapped reads	37,916,914 (83.93%)	38,678,230 (90.13%)	46,687,041 (89.43%)	48,971,691 (88.94%)	32,192,365 (87.97%)	40,452,633 (88.56%)
Multiple mapped reads	4,393,614 (9.73%)	2,729,634 (6.36%)	3,882,395 (7.44%)	4,178,505 (7.59%)	2,796,339 (7.64%)	3,474,003 (7.61%)

## Data Availability

The RNA sequence raw data were deposited in the China National Center for Bioinformation (CNCB) Genome Sequence Archive (GSA; accession number CRA049220) at https://ngdc.cncb.ac.cn/gsa/browse/CRA049220, accessed on 28 April 2026.

## Supplementary Materials

The following supporting information can be downloaded at https://www.mdpi.com/article/10.3390/biology15191731/s1, Table S1: Gene-specific primers used for RT-qPCR analysis; Table S2: Differentially expressed genes (DEGs) in *Bombyx mori* larvae induced by zinc iron oxide; Table S3: The 18 most enriched KEGG pathways ranked by *p*-value for all differentially expressed genes in *Bombyx mori* larvae induced by zinc iron oxide.

## Abbreviations

The following abbreviations are used in this manuscript:

ZIO	Zin Iron Oxide
ROS	Reactive Oxygen Species
SOD	Superoxide Dismutase
CAT	Catalase
GSH	Glutathione
DEGs	Differentially Expressed Genes
KEGG	Kyoto Encyclopedia of Genes and Genomes
